# Supplementary figures and images for: Component of nicotine-induced intracellular calcium elevation mediated through α3- and α5-containing nicotinic acetylcholine receptors are regulated by cyclic AMP in SH-SY 5Y cells
Source: PLoS One. 2020 Nov 30;15(11):e0242349. doi: 10.1371/journal.pone.0242349 (PMC7703979; doi:10.1371/journal.pone.0242349)

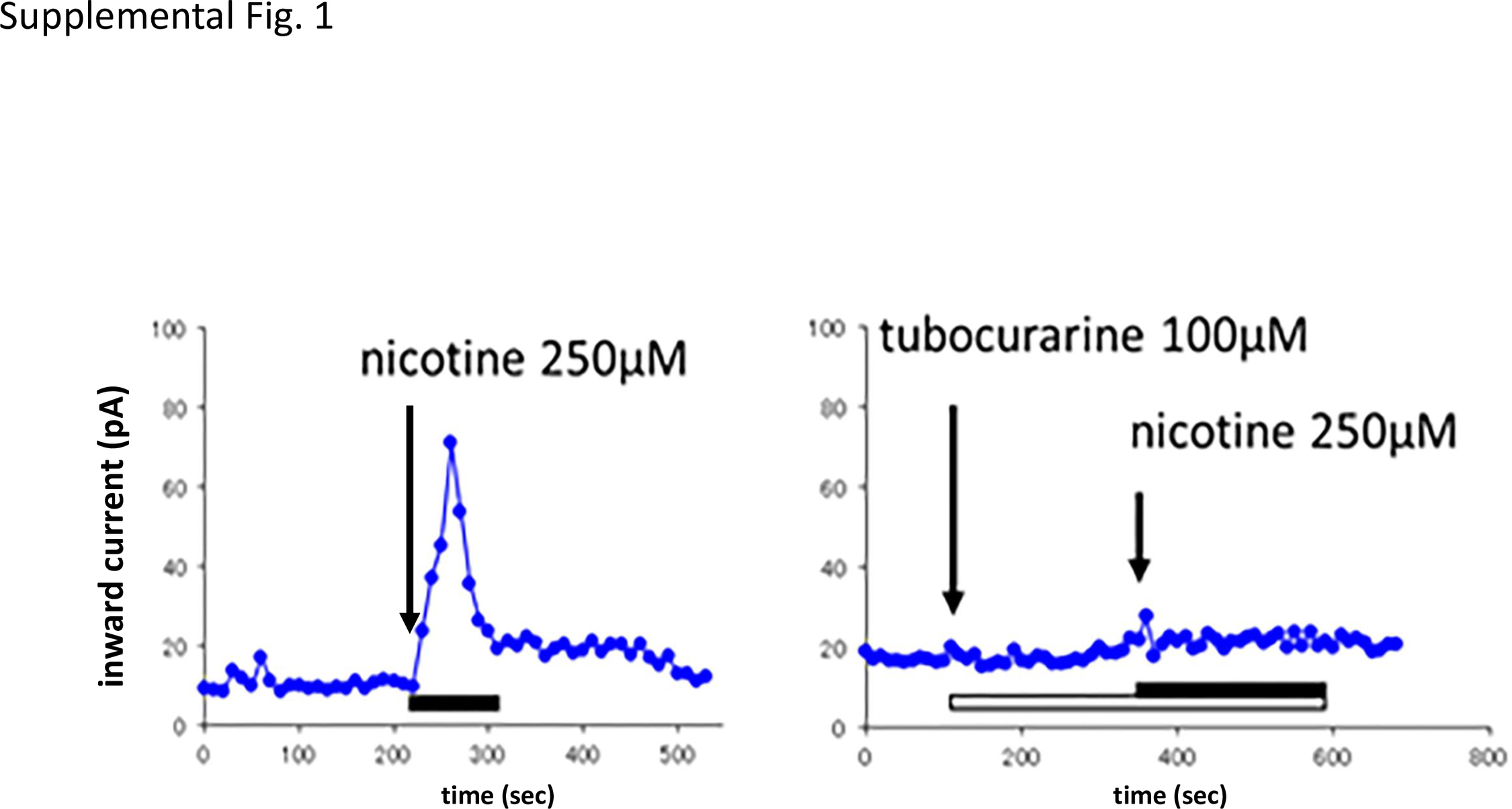

Supplement: S1 Fig — The representative time plot of leak currents at -70 mV. Leak currents were sampled every ten seconds. Left panel: The bath application of nicotine (250 μM) evoked the large inward current in an SH-SY5Y cell. Nicotine was applied during a period indicated by a black line. Right panel: Pretreatment with 100 μM tubocurarine (white line) completely suppressed the nicotine-induced current. Representative data from 3experiments are presented. (TIF) [file pone.0242349.s001.tif]
