# Supplementary material for: Component of nicotine-induced intracellular calcium elevation mediated through α3- and α5-containing nicotinic acetylcholine receptors are regulated by cyclic AMP in SH-SY 5Y cells
Source: PLoS One. 2020 Nov 30;15(11):e0242349. doi: 10.1371/journal.pone.0242349 (PMC7703979; doi:10.1371/journal.pone.0242349)
Supplement: Attachment — Submitted filename: Reply to reviewer.docx [file pone.0242349.s002.docx]

Overall revision

We have added two authors, Takayuki Yoshida and Kana Harada, who did the experiments for revising manuscript.

Reply to reviewer #1

We appreciate the comments that lead to the improvement of this manuscript.

1) In section 2-3, effect of tubocurarine was tested on [Ca2+]i levels of cells treated with 250 µM nicotine. A complete inhibition of inward currents was observed while [Ca2+]i was significantly reduced but not complete. Could the authors comment on why 250 µM nicotine was used rather than 500 µM. This is important since (a) in Section 2-2 (Fig. 2), to check the source of [Ca2+]i the authors used 500 µM nicotine and (b) effect of 500 µM nicotine on [Ca2+]i (Fig. 1B) was much more pronounced compared to 250 µM. Verifying the effect of 500 µM nicotine is therefore important, especially on the current.

According to the reviewer’s comment, we investigated the effects of tubocurarine on the currents induced by 500 μM nicotine. As shown in revised figure 3A, 100 μM tubocurarine almost completely suppressed the current induced by 500 μM nicotine as well as the currents induced by 250 μM nicotine as shown in a former figure 3A. In addition, quantitative analysis of tubocurarine effects was added as Figure 3B.

A former figure 3A showing the effect of tubocurarine on the 250 μM nicotine-induced current has been presented as a supplemental figure 1.

We have observed nicotine-induced calcium elevation at concentrations up to 500 μM. Generally, pharmacological studies using inhibitors and siRNAs are usually performed at intermediate concentrations (preferably at ED50 concentrations) rather than at the highest concentrations tested. Therefore, in this study, we examine the effects of various inhibitors and siRNAs on nicotine-induced calcium elevation at the concentration of 250 μM. However, in the experiments shown in Figure 2, we had to observe a significant nicotine-induced calcium elevation under various conditions, so we used 500 μM nicotine, at the highest concentration of nicotine tested.

The description in the main text has been changed in accordance with the change in Figure 3 as follows.

Material methods section; page 7, line 16- page 8, line 1, page 8, line6-7

Results section; page 10, line18-24

Figure legends; page 25, line 11-20

2) Based on Fig. 1A, expression of α7 in SHSY-5Y cells is significantly lower than α3 and α5, which raises the question whether this cell line is an ideal system for studying the involvement of α7nAChR in regulating [Ca2+]i levels.

In this study, the mRNA expression of each nAChR in SHSY5Y cells was examined only once. Therefore, three new cases were examined and the results was presented as a new Figure 1A. Again, α7 expression appears to be lower than α3 and α5 expressions in the graph of new figure 1A. However, the efficiency of the primers to detect each nAChR subunit and the efficiency of the cDNA synthesis of each nAChR subunit in the process of synthesizing cDNA from mRNA are not same between each subunit. Therefore, we cannot simply judge from this graph that the expression of α7 is lower than those of α3 and α5.

A PubMed search yielded 88 papers investigating nicotine-induced calcium elevation via the α7 nAChR in SHSY-5Y cells. This fact does not suggest that SHSY-5Y cells are not ideal for studying nicotine-induced calcium elevation via the α7 nAChR.

Grammatical errors / typos:

1) Typo in the title of the article “though” should be replaced by “through”.

2) Page 12, Line 4 – Fig. 4E has been mistakenly typed as Fig. 2E

We corrected these errors in the revised manuscript.

Reply to reviewer #2

We appreciate the comments that lead to the improvement of this manuscript.

-Page 3, Line 23: The authors should define “Gs” before using the abbreviation here or elsewhere.

We have changed the description “Gs” to “Gs (stimulatory G protein)”. (page 3, line 23-24)

- Page 5, Line 24: For real-time qPCR using the Livak method (2^-deltadelta Ct), the authors should follow the MIQE (Minimum Information required for publication of Q-PCR Experiments) guidelines (Bustin et al. (2009), Clin Chem, 55:611-622) by providing the primer efficiencies and R-squared of the PCR.

We noticed that the delta Ct method was used in this study, rather than the 2^-deltadelta Ct method, to assess mRNA expression, so we have changed the description to reflect that. Information for the primer efficiencies and R-squared of the PCR have been also described in revised manuscript. (page 6, line 6-9)

And, we re-performed the RT-PCR studies three times because the experiment was done only once in a former manuscript. The new results have been presented as figure 1A.

- Page 7, Line 13: The authors should indicate how many independent trials were done, and how many cells were voltage-clamped to arrive with the data they presented on Fig. 3A.

We newly investigated the effect of tubocurarine on the current induced by 500 μM nicotine. We performed 8 independent experiments. The descriptions were changed accordingly.

Material methods section; page 7, line 16- page 8, line 1, page 8, line6-7

Results section; page 10, line18-24

Figure legends; page 25, line 11-20

- Pages 24-28, Figure Legends: The authors should consistently indicate or mention the independent trials or cell numbers if applicable. For example, on figure 1, the legend does not provide experimental trials for the left panel of Fig. 1B, but they show on the right panel n=6 for 100 uM, n=4 for 250 uM, n=4 for 500 uM). It is important to mention the trials every time especially that they mentioned that the data are represented as means +/- SEM.

In accordance with the reviewer's suggestion, we have added a description concerning the number of experiments and the representation of the data. (page 24, line 5-6, line 11, page 25, line 4-5, page 25, line 15, page 25, line 24)

With regard to data representation, we have already described them as “Data represent the means ± standard error”.

-Also, the same issue applies in other figures where the authors showed %fluorescence or trace, but they did not mention on the Fig Legend the experimental trials done.

Concerning the trace using %fluorescence, we have already described the information in materials methods and figure legends section as bellow.

The time-dependent change in intracellular calcium ion [Ca^2+^]i was determined by setting the fluorescence intensity to 100% before administration of nicotine or acetylcholine. (page 6, line23 -page 7, line 1)

The fluorescence before nicotine administration was assumed to be 100%, and the change in [Ca^2+^]i after application of nicotine at each concentration is shown ( page 24, line 9-10)
